# The effect of access to electronic resources during examination on medical and dental students scores in summative assessment: quasi experimental study

**DOI:** 10.1186/s12909-022-03904-8

**Published:** 2022-12-12

**Authors:** Shatha Al-Sharbatti, Hossam Hamdy, Salah Eldin Kassab, Manda Venkatramana

**Affiliations:** 1 Community Medicine Department, College of Medicine, Gulf Medical University, Ajman, UAE; 2 Department of Physiology, Faculty of Medicine, Suez Canal University, Ismailia, Egypt; 3 Surgery and Medical Education, Gulf Medical University, Ajman, UAE; 4 Physiology and Medical Education, Gulf Medical University, Ajman, UAE; 5 Consultant Surgeon, Thumbay Hospital, Ajman, UAE; 6 Gulf Medical University, Ajman, UAE

**Keywords:** E-resources, Score, Test, Cognitive-level, Students

## Abstract

**Background:**

Access to electronic (E) resources has become an indispensable requirement in medical education and practice.

**Objective:**

Our objective was to assess the effect of E-resources access during examination on end-course-exam scores of medical and dental students.

**Methods:**

A quasi-experimental study which included two cohorts of medical (*n* = 106 & 85) and three cohorts of dental students (*n* = 66, 64 and 69) who took end-course- exams. Each exam was composed of two- parts (Part I and II), that encompassed equal number of questions and duration. Access to E-resources was allowed in part-II only. Items Difficulty Index (DI), Discrimination Index, (DisI), Point Biserial, (PBS) and cognitive level were determined.

**Results:**

The study included 390 students. The proportion of items at various levels of DI, DisI, and PBS and the average values for item DI, DisI in both parts of each exam were comparable. The average scores in part-II were significantly higher than part-I (*P* < 0.001, < 0.001 and 0.04) and lower-order cognitive-level items scores were higher in three exams (*P* < 0.0001, 0.0001, 0.0001). Higher- order cognitive level items scores were comparable between part I and II in all courses. The significant factor for change in marks were questions cognitive level and type of the course.

**Conclusion:**

Access to E-resources during examination does not make a significant difference in scores of higher-order cognitive level items. Question cognitive level and course type were the significant factors for the change in exam scores when accessing E-resources. Time-restricted E-resources accessed tests that examine higher cognitive level item had no significant academic integrity drawback.

## Background

Electronic (E) resources include various internet-based sources of videos, lectures, textbooks, flashcards, MCQs, online modules, online publications, and conferences [1]. Studies documented the value and effectiveness of accessing E-resources on student learning. A cross-sectional study included 231 students from five health colleges taking a common physiology course during their second year. The study reported a significant correlation between the use of technology by students and their academic achievements [2]. Similarly, another study that included students from four universities in KSA reported associations between the adoption of E-resources in learning and students’ academic performance [3].

Despite the reported usefulness of accessing E-resources for learning, studies that investigated the impact of accessing resources during the exam on students’ performance have yielded controversial results. The resources-accessed test is commonly referred to as an open-book test (OB) to denote an assessment method that permits a textbook or notebook, or any reference materials during the examination. At the same time, a closed-book test (CB) is a test in which the students are not allowed to consult their material or resources [4]. The examination context can be classroom or electronic; both contexts can be with or without invigilation, tight-time restriction, or a less restricted time window [5]. It is assumed that giving access to e-resources during the test will inflate the marks, although the literature showed variable findings. Durning et al., conducted a systematic review to investigate the change in students’ performance who participated in open-book and closed-book exams [6]. The authors had reviewed 37 studies and found no difference in examinee scores between OB and CB exams in most of the reviewed studies. In the same vein, educators compared student achievement on the usual closed-book knowledge-based test with performance on the open internet competency-based information mastery assessment (IMA) in Family Medicine Clerkship Exam [7]. The author found that student scores on the internet-accessed IMA were comparable to that from the closed-book knowledge-based test. A similar finding was also obtained in another study that compared the mark of students in online invigilated and non-invigilated tests after controlling for the GPA of students [8]. The authors found no significant differences between the mean scores of students in the two groups of students. Rummer et al. compared OB and CB testing in two parallel university courses and found that participants in the CB tests had better performance [9]. The previously mentioned findings differ from a study on 274 students enrolled in a psychology course which demonstrated that students scored significantly higher on the un-proctored online tests than their peers who had taken the classroom-based, proctored, paper and pencil tests [10]. Similarly, a study from Thailand compared the 4th- year medical students’ scores in online, open-book surgery clerkship test scores with the usually written tests. The authors found that students who took the open-book tests had significantly higher mean scores for multiple-choice and essay questions [11]. Likewise, a study compared the differences between students’ scores in OB and CB exams in different types of psychology courses. The authors found that students did better on OB exams [12]. The previous review showed inconsistent findings in relation to the impact of resource access on students’ scores. Researchers argued about the need for assessment formats that simulate real-life access to E-resources while considering the general concern against that access in relation to the possible inflation of the marks [8, 13].

In this study, we investigate the impact of E-resources access during the exam on scores of the same cohorts of students who had recently taken CB exam format for the same course. We have also compared the item analysis characteristics in exams with and without access to E-resources. The study hypothesized no significant difference in scores when students access the E-resources during the exam.

## Methods

### The objectives

To assess the effect of E-resources access during examination on end-course-exam scores of medical and dental students.

### Design

A quasi-experimental study.

### The setting of the study

Gulf Medical University (GMU).

### The characteristics of participants

The study included two cohorts of medical students (*n* = 106 & 85) and three cohorts of dental students (*n* = 66, 64 and 69) who took end-course exams (total = 390). Medical students included those who took the End-course exam for Respiratory System (*n* = 106) and End of Clerkship Phase exam (*n* = 85). Dental students included those who have taken end-course exams in Oral Surgery (*n* = 66), Orthodontics − 2 (*n* = 64) and Exit DMD Program exam (*n* = 69). The study excludes students who had not taken part in the selected exams.

### Methods

Each test is composed of two parts (part-I and part-II**)** that encompass an equal number of type A-multiple choice questions (MCQs), equal time, computer-based, invigilated and took place on campus. The E-resources access was denied in part-I and allowed in part-II of each exam. The study included 370 test items. Verification of item cognitive levels was provided by at least two faculties for each course. Items were identified to assess two cognitive levels, namely higher (e.g., application) or lower (recall/ comprehension), according to Bloom’s taxonomy. Cohen’s kappa coefficient was used to measure inter-rater reliability. Kappa Coefficient values of 0.9 and more were obtained for all courses. The consensus of the faculty about the cognitive level of the items was obtained. The average scores of each student in part I and part II of each test by the cognitive level of items and access to E-resources were determined and compared. Students received an equal proportion of questions in the two selected cognitive levels in part I and part II of each test. The E-resources available to the students were library E-resources, Moodle learning platform, scientific E- resources web search (Scholar Rx, Google Scholar, PubMed,.), and nonspecific google search.

To assess the comparability of test items that were given in part I and part II, the Quality indicators of test items [Items Difficulty Index (DI), Discrimination Index, (DisI), Point Biserial, (PBS) and cognitive level] and Test Reliability (KR-20) were examined and compared [14, 15].

The student’s marks for part 1 and part II and the cumulative GPA(CGPA) values were also compared. A post-test survey was done. The students were asked to reflect if they expect a higher score on E-resource accessed exams and if they think they are prepared well for the exams.

### Statistical analysis

Data analysis used the SPSS version-27. The differences between scores of each student in the two parts of the test and for each cognitive level questions were assessed using paired Students’ t-test. The differences between the mean values of DI, and DisI in part-I and part-II were assessed using an independent sample t-test (pooled variance). The chi-square test was used to test the association between item acceptability and access to E-resources as well as the association between CGPA, perceived score, and perceived efficacy and change in the marks on accessing E-resources.

### Ethical considerations

The university Institution Review Board (IRB) approved the study *[Ref.no.IRB/MHPE/STD/19/Dec-2020]*, waiver the requirement of informed consent was obtained. The students had been informed about the structure of the test before taking the test. Confidentiality of the information was respected and only the research team and the-IRB can access the data. Analysis was done groupwise, so there is no link between result and any participant in person. All methods were performed in accordance with the relevant guidelines and regulations.

## Results

The study included 390 students and 370 items. The number of participating students (NS) and the number of items (NI) given to them were the same in e- resource accessed and denied parts of each test, and they were as follows: Oral Surgery (NS = 66, NI = 30), Orthodontics (NS = 64, NI = 30), Exit DMD (NS = 69, NI = 50), Respiratory System (NS = 106, NI = 25), and Exit MBBS (NS = 85, NI = 50). Asking participants about the most commonly accessed E- resources during the E- resource accessed tests showed that 27.2% (*n* = 106) of the participants had done a nonspecific google search and in 46.5% (*n* = 174) Moodle search was done, while 46.8% of students (*n* = 174) reported searching scientific website (PubMed, Google Scholar, Scholar Rx,.) and Library E-resources (19.8%, *n* = 74), respectively.

Most students expected to receive a higher score on the online accessed test (69.1%), and 66.8% of them perceived that they were well prepared for the test (perceived efficacy). Test reliability values were within the acceptable level (≥ 0.70) for both parts of each test.

Scores of students in the E-resource- accessed and E-resource- denied tests by courses were given in Table 1. which showed that in three courses, the average mark of students was significantly higher when they had accessed E- resources. Overall analysis showed a significant increase in marks on accessing E-resources during examinations. There was a wide confidence interval indicating variability in students’ performance with and without accessing E-resources.

**Table 1:** The Students’ Scores in the E-Resource- Accessed and Denied Tests by Courses

Course	Number	Students’ Scores				P*
		No Access To E-Resources		Access To E-Resources		
		Mean	SD	Mean	SD	
Oral Surgery	66	56.8	14.8	61.9	14.6	< 0.001
Orthodontics	64	65.4	17.4	66.6	17.1	0.356
Exit DMD	69	62.3	15.2	60.8	15.9	0.232
Respiratory System	106	50.0	19.0	57.4	15.2	< 0.001
Exit MBBS	85	58.4	15.1	60.3	13.6	0.044
Total	390	57.7	17.4	63.3	49.9	0.024

*Paired t-test

We compared the test quality indicators of the two parts of each test to exclude the possibility that the change in scores was due to differences in test items’ quality.

The number (%) of items that were within acceptable PBS level (i.e.: PBS ≥ 0.15) were 169 (91.4%) and 165 (89.2%) in E-resource accessed and denied tests (*p* = 0.483).

The mean (SD) of ID, DisI and PBS in E-resources accessed and denied tests were 0.59 (0.21) and 0.61(0.21); 0.39(0.20) and 0.36(0.18); 0.81 (0.04) and 0.78(0.07) respectively. No significant differences between the mean values of the test quality indicators of the two parts of each test (P > 0.05 for all indicators).

Table 2. Showed the average scores of items testing lower and higher cognitive level orders with and without access to E -resources across tests. The table showed no significant differences between average scores for higher cognitive level order items; however, differences were noticed between the average scores of lower cognitive level items for three tests. It can be suggested that the significant difference earlier noticed between scores of students in three tests conducted with and without having access to E -resources were developed from items assessing lower cognitive level competence.

**Table 2:** The Scores of Students by Courses, Cognitive level and Access to E- Resources

Course	Access to E resources Status	Cognitive level	Number of students	Mean Marks	SD	P*
Oral Surgery	No	Lower	66	56.8	20.8	< 0.0001
	Yes	Lower	66	65.7	14.4	
	No	Higher	66	56.8	13.9	0.697
	Yes	Higher	66	57.6	18.3	
Orthodontics	No	Lower	64	75.1	17.9	0.586
	Yes	Lower	64	76.3	17.9	
	No	Higher	64	55.7	20.9	0.345
	Yes	Higher	64	58.0	18.7	
DMD Exit Exam	No	Lower	69	57.9	17.2	0.442
	Yes	Lower	69	56.5	17.9	
	No	Higher	69	64.1	15.8	0.442
	Yes	Higher	69	63.5	16.6	
MED 204	No	Lower	106	50.4	19.7	< 0.0001
	Yes	Lower	106	68.6	18.2	
	No	Higher	106	49.6	22.9	0.412
	Yes	Higher	106	51.1	17.3	
MBBS Exit Exam	No	Lower	85	78.8	19.2	< 0.0001
	Yes	Lower	85	86.5	14.5	
	No	Higher	85	55.8	15.7	0.112
	Yes	Higher	85	53.8	14.5	

*Paired t-test

Table 3 showed that the change in marks on accessing E - resources was not associated with CGPA, perceived higher score, and perceived efficacy and change in the marks on having access to E - resources.

**Table 3:** The association between CGPA*, perceived higher score, and perceived self-efficacy and change in the marks on having access to E-resources

Variable	Subgroup	Marks Change						*P*
		No difference		Increased		Decreased		
		No	%	No	%	No	%	
CGPA*	< 2.0	0	0.0	3	75.0	1	25.0	0.549
	2-2.4	2	18.2	5	45.5	4	36.4	
	2.5–2.9	4	9.5	24	57.1	14	33.3	
	3-3.4	8	10.3	40	51.3	30	38.5	
	3.5–3.9	11	10.7	50	48.5	42	40.8	
	4.0	9	12.0	49	65.3	17	22.7	
Perceived higher score	No	9	7.9	61	53.5	44	38.6	0.344
	Yes	30	11.8	142	55.9	82	32.3	
Perceived self-efficacy	No	13	10.5	66	53.2	45	36.3	0.884
	Yes	26	10.6	137	55.7	83	33.7	

*CGPA Cumulative Grade Point Average

## Discussion

There is a growing use of online resources in medical education and practice [16]. The current work studied factors that may explain the differences in marks of students on accessing E-resources.

In the present study, the average scores in three E-resource-accessed exams were significantly higher than E -resource denied exams. This finding is consistent with that developed by some researchers. It should be noted that most of the previous studies compared the marks across two different cohorts who had received access to resources (non-proctored) with that in face-to-face tests without examining the characteristics of the test items. Some researchers found no difference in marks [7, 17, 18]. Other researchers found higher marks among students who took the online test [11, 12, 19]. Anaya, et al. [20] had mixed results, with students in face-to-face tests attaining higher scores for some classes and lower scores for the other classes, and overall results were not significant. A study conducted by Eurboonyanun et al. [11] showed an increase in the average scores of students who had taken online exams during COVID 19 compared with the average scores for their previous face-to-face tests. The authors in the previous study had not examined the items’ cognitive levels and couldn’t explain the observed difference.

Soto Rodríguez et al. [21] compared “the percentile ranking scores” of two cohorts received in end-of-course “computer-based” and “paper-based” assessments. The authors found that pupils were ranked ordered equivalently at the cohort level in both testing modes. The authors suggested that this way of comparing the two exam modes can overcome the possible impact of differences on the testing experience in the two test modes.

A previous systematic review that investigated the difference in students’ performance on having access to e-resources found a similar performance of students in most of the reviewed studies, and in two studies, the performance was better in CB exam [6]. This finding disagrees with the common expectation that examinees would perform better in OB exams because they can look up answers. The authors presume that students invest more time and effort in preparing for CB exams than OB exams. We want to note here that in the current study, there was no difference in the exam preparation time for part-I and part-II of each test because both parts were conducted on the same day, and the variabilities in test preparation time reflect the choice of learners and cannot explain the difference in students’ scores in these tests. Moreover, the two parts of each exam (with and without access to E-resources) were taken by the same students, which helped overcome possible individual differences that may affect the scores in both parts.

In the current study, we examined the test item characteristics to find explanations for the observed differences in the marks in some tests. Item analysis assists in determining items “to retain, modify or discard” for a particular exam [22].

Most of the items had acceptable discriminating levels in our study, and only 11% had a poor discrimination index. This percentage is lower than the item analysis done by Kaur et al. [23]

The present data showed that the average items difficulty index, discrimination index, and point biserial in E-resource accessed Part-II and E-resource denied Part-I of all tests were comparable. This finding differs from Lipner et al. [24] trial that examined the effect of E-resources access on the performance of physicians who sat for a test that mirrored the Internal Medicine Maintenance of Certification (IM-MOC) examination. The authors compared item difficulty in the two parts of the trial and found that the mean discrimination was statistically significantly higher for open-book conditions.

Few studies analyzed the scores based on the cognitive level of the test items. This study showed that the increase in marks only existed for lower cognitive level items. This finding underscores the importance of well-formulated test items. The results of this study support the guides provided by the University of New South Wales [25] to use higher cognitive level questions that test and prob critical reasoning rather than recall, which can encourage cheating.

Sam et al., study compared the psychometric analyses of the open-book (OB) exams used to assess final-year medical students during the early COVID- 19 pandemic with those of the written closed-book (CB) exams that were used for another cohort of students in the previous three years. The authors found that the average values for DisI and PBS were comparable. The authors suggested that access to OB resources didn’t systematically affect student performance [26].

Davies et al. compared the performance of second-year medical students on summative end-of-year OB exam with marks obtained by another cohort of students who had taken the exam for the same course but with a different set of questions in the previous year in CB format. Authors in the previous study examined the questions’ cognitive level, which was categorized into- two levels of Blooms’ categories. The authors found that access to OB resources was associated with higher scores for both higher and lower cognitive level items, with a greater difference for recall items compared to understand/apply questions [27]. The later finding disagrees with our results which showed differences in only low cognitive level questions.

It should be noted that in the previous studies, the authors compared the scores of students from different cohorts and/ or at different times, which raises the doubt that personal factors can be a source of bias in these studies. We tried our best to make the two parts of the tests comparable in all controllable factors to increase the validity of the results. To the best of our knowledge, this is the first time to compare the score of the same cohort of students who took part in end-course tests that allow them to have the two-test format with and without access to E-resources during the examination, thus to control for the possible sources of bias that can emerge from comparing different cohort, different times, different course materials and different quality of questions. One important finding in our study is that even though psychometric properties of tests are valuable for defining the reliability and quality of the test, however, they have limited values in reflecting the cognitive levels of the construct. The latter is very important in selecting questions for resource-accessed tests. In addition, despite the presence of much evidence supporting the use of this modality as one approach to testing students’ performance, many educators are hesitating because of the concern that it may lead to an inflation of the marks, which will affect the integrity of the test. Our study showed that the problem is not with the approach, it is with the questions. If recall questions are given, students can easily find the answer. However, if high cognitive level questions require the application of knowledge, with limited time, a scenario similar to what doctors usually face in everyday practice, only those students who know what information they need and how to do a proper search will benefit from that access. Thus, it can be assumed that using E-resource-accessed tests allow the assessment of higher-order cognitive knowledge and stimulate scientific search competencies required by future doctors.

In our study, we found no association between CGPA, and change in the marks on having access to e-resources. This result is supported by Rani et al. [28] found that the academic performance of undergraduate medical students was not associated with the change in marks on accessing E-resources. We also noticed that although most of the participants perceived self-efficacy and that they were well prepared for the exam; however, this positive belief was not associated with an increase in their marks, and almost half of the students (44%) had either decreased or no change in the mark. The latter finding disagrees with Kitsantas, et al. [29], who found that perceived self-efficacy can predict academic achievement.

## Conclusion

Access to E-resources during examinations does not make a significant difference in scores of higher-order cognitive level items. Question cognitive level and course type were the significant factors for the change in exam scores when accessing E-resources. Time-restricted E-resources accessed tests that examine higher cognitive level items had no significant academic integrity drawback.

### Limitation of the study

The wide standard deviation of average students’ scores indicated variability and reduced the precision of the calculated values. It should be noted that the assessment of quality indicators showed that test items used were within an acceptable level, which indicated that the mentioned scores variability is possibly related to personal factors of the studied population.

### Recommendation

The present findings raise important needs for a critical selection of items in online accessed tests. Future studies are recommended to explore the psychological impact of accessing E-resources during the exam on students.

## Data Availability

The datasets used and/or analysed during the current study are available from the corresponding author on reasonable request.

## References

[1] Schneider M, Binder T (2019). E-Learning in medicine: current status and future developments. Hamdan Med J.

[2] Al-Hariri M, Al-Hattami A (2017). Impact of students’ use of technology on their learning achievements in physiology courses at the University of Dammam. J Taibah Univ Med Sci.

[3] Basri WS, Alandejani JA, Al-Madani FM. ICT adoption impact on students’ academic performance: evidence from saudi universities. Educ Res Int, 2018. P: 1–9. 10.1155/2018/1240197.

[4] Goothy SSK, Suphal S, Bandaru TS (2019). Comparison of academic performance and stress levels in open book test and closed book test and perceptions of undergraduate dental students. MOJ Anat & Physiol.

[5] Myyry L, Joutsenvirta T (2015). Open-book, open-web online examinations: developing examination practices to support university students’ learning and self-efficacy. Act Learn High Educ.

[6] Durning SJ, Dong T, Ratcliffe T, Schuwirth L, Artino AR, Boulet JR, Eva K (2016). Comparing Open-Book and Closed-Book Examinations: A Systematic Review. Acad Med..

[7] Erlich D. Because life is open book: an open internet family medicine clerkship exam. PRiMER. 2017;1,7. Available from: https://journals.stfm.org/primer/2017/erlich-2016-0011/. 10.22454/PRiMER.2017.626578 PMC7490191 32944693

[8] Hollister KK, Berenson ML (2009). Proctored Versus Unproctored Online Exams: studying the impact of exam environment on Student Performance. Decis Sci J Innovative Educ.

[9] Rummer R, Schweppe J, Schwede A (2019). Open-book Versus closed-book tests in University classes: a field experiment. Front Psychol..

[10] Brallier S, Schwanz KA, Palm L, Irwin L (2015). Online testing: comparison of Online and Classroom Exams in an Upper-Level psychology course. Am J Educational Res.

[11] Eurboonyanun C, Wittayapairoch J, Aphinives P, Petrusa E, Gee DW, Phitayakorn R (2020). Adaptation to Open-Book Online Examination during the COVID-19 pandemic. J Surg Educ.

[12] Gharib A, Phillips W, Mathew N (2012). Cheat sheet or Open-Book? A comparison of the Effects of exam types on performance, Retention, and anxiety. J Psychol Res.

[13] Fuller R, Joynes V, Cooper J, Boursicot K, Roberts T (2020). Could COVID-19 be our ‘there is no alternative’ (TINA) opportunity to enhance assessment?. Med teach..

[14] Rudolph MJ, Daugherty KK, Ray ME, Shuford VP, Lebovitz L, DiVall MV (2019). Best Practices related to examination Item Construction and Post-hoc Review. Am J Pharm Educ.

[15] Sharma LR (2021). Analysis of Difficulty Index, discrimination index and distractor efficiency of multiple choice questions of Speech Sounds of English. Int Res J MMC.

[16] Serdyukov P (2017). Innovation in education: what works, what doesn’t, and what to do about it?. J Res Innovative Teach Learn.

[17] Frein S (2011). Comparing in-class and out-of class computer-based tests to traditional paper-and-pencil tests in introductory psychology courses. Teach Psychol.

[18] Paul J, Jefferson FA (2019). Comparative analysis of Student Performance in an online vs. face-to-Face Environmental Science Course from 2009 to 2016. Front Comput Sci.

[19] India L, Broyles, Peggy R, Cyr (2005). Neil Korsen. Open book tests: assessment of academic learning in clerkships. Med Teach.

[20] Anaya LH, Evangelopoulos N, Lawani U. Open Book Vs. Closed Book Testing: An Experimental Comparison. June 20–23, 2010, Annual Conference & Exposition. URL: https://strategy.asee.org/collections/15.

[21] Soto Rodríguez EA, Fernández Vilas A, Díaz Redondo RP (2021). Impact of computer-based assessments on the Science’s ranks of secondary students. Appl Sci.

[22] Shigli K, Nayak SS, Gali S, Sankeshwari B, Fulari D, Shyam Kishore K, Upadhya P, Jirge V (2018). Are multiple choice questions for Post Graduate Dental Entrance Examinations Spot on?-Item analysis of MCQs in Prosthodontics in India. J Natl Med Assoc.

[23] Kaur M, Singla S, Mahajan R (2016). Item analysis of in use multiple choice questions in pharmacology. Int J Appl basic Med Res.

[24] Lipner RS, Brossman BG, Samonte KM, Durning SJ (2017). Effect of Access to an Electronic Medical Resource on performance characteristics of a certification examination: a Randomized Controlled Trial. Ann Intern Med.

[25] University of New South Wales (UNSW). Why do online open-book exams deserve a place in your course? Sydney, Australia. May2020. Available at: https://www.education.unsw.edu.au/news-events/news/why-do-online-open-book-exams-deserve-a-place-in-your-course.

[26] Sam AH, Reid MD, Amin A (2020). High-stakes, remote-access, open-book examinations. Med Educ.

[27] Davies DJ, McLean PF, Kemp PR, Liddle AD, Morrell MJ, Halse O, Martin NM, Sam AH (2022). Assessment of factual recall and higher-order cognitive domains in an open-book medical school examination. Adv Health Sci Educ Theory Pract.

[28] Rani V, Singh NP, Bharti PP. Factors Affecting the Academic Performance of Undergraduate Medical Students at a Medical Institute of Northern India. J Med Educ Dev. 2021; 13 (39):1–9. URL: http://zums.ac.ir/edujournal/article-1-1343-en.html.

[29] Kitsantas A, Cheema J, Ware HW (2011). Mathematics achievement: the role of homework and self-efficacy beliefs. J Adv Acad.

